# Online Alcohol Assessment and Feedback for Hazardous and Harmful Drinkers: Findings From the AMADEUS-2 Randomized Controlled Trial of Routine Practice in Swedish Universities

**DOI:** 10.2196/jmir.4020

**Published:** 2015-07-09

**Authors:** Preben Bendtsen, Marcus Bendtsen, Nadine Karlsson, Ian R White, Jim McCambridge

**Affiliations:** ^1^ Medical Faculty Department of Medical Specialist and Department of Medicine and Health Sciences Linköping University, Motala Linköping Sweden; ^2^ Technical Faculty Department of Computer and Information Science Linköping University Linköping Sweden; ^3^ Medical Faculty Department of Medicine and Health Sciences Linköping University Linköping Sweden; ^4^ MRC Biostatistics Unit Cambridge Institute of Public Health Cambridge United Kingdom; ^5^ Faculty of Science Department of Health Sciences Universiyt of York Heslington United Kingdom

**Keywords:** alcohol drinking, behavior therapy, students, Internet, electronic mail, feedback

## Abstract

**Background:**

Previous research on the effectiveness of online alcohol interventions for college students has shown mixed results. Small benefits have been found in some studies and because online interventions are inexpensive and possible to implement on a large scale, there is a need for further study.

**Objective:**

This study evaluated the effectiveness of national provision of a brief online alcohol intervention for students in Sweden.

**Methods:**

Risky drinkers at 9 colleges and universities in Sweden were invited by mail and identified using a single screening question. These students (N=1605) gave consent and were randomized into a 2-arm parallel group randomized controlled trial consisting of immediate or delayed access to a fully automated online assessment and intervention with personalized feedback.

**Results:**

After 2 months, there was no strong evidence of effectiveness with no statistically significant differences in the planned analyses, although there were some indication of possible benefit in sensitivity analyses suggesting an intervention effect of a 10% reduction (95% CI –30% to 10%) in total weekly alcohol consumption. Also, differences in effect sizes between universities were seen with participants from a major university (n=365) reducing their weekly alcohol consumption by 14% (95% CI –23% to –4%). However, lower recruitment than planned and differential attrition in the intervention and control group (49% vs 68%) complicated interpretation of the outcome data.

**Conclusions:**

Any effects of current national provision are likely to be small and further research and development work is needed to enhance effectiveness.

**Trial Registration:**

International Standard Randomized Controlled Trial Number (ISRCTN): 02335307; http://www.isrctn.com/ISRCTN02335307 (Archived by WebCite at http://www.webcitation.org/6ZdPUh0R4).

## Introduction

College and university students in Sweden, as in other parts of the world, drink alcohol heavily [1-2]. Because alcohol is responsible for substantial adverse health consequences and social problems [3-4], there is a need for effective interventions. Swedish universities offer preventive services aiming at reducing drinking among students through 26 local student health care centers nationally. Human resources (ie, staff numbers) are not sufficient to offer face-to-face brief interventions to all risky (hazardous) or problem (harmful) drinkers, although such interventions have been shown to have small beneficial effects [5-6]. Even if offered, take-up can be expected to be low and the Internet offers promise for wider-reaching and cost-effective interventions [6,7].

Existing evidence of the effectiveness of online interventions with students is mixed [6-8] partly because of unresolved methodological challenges including attrition prevention and assessment reactivity [9]. In a narrative review in 2008 on online alcohol interventions targeting students, Elliott et al [10] found an effect that was better than no intervention and equivalent with other alcohol interventions. In another review in 2009, Carey et al [6] reviewed the effect of online alcohol intervention including 26 studies targeting students and found an overall short-term reduction in alcohol consumption with weighted mean effect sizes for various alcohol measures from approximately 0.13 to 0.29 that decreased over time. A significant variability in efficacy was seen due to a heterogeneity of content, tailoring, and method of access to the intervention (ie, logging on to a website on home computer or performing the intervention in an office-based setting) [6]. In a later meta-analysis in 2009 of 43 online interventions to student populations, the interventions were found to reduce both quantity and frequency measures of consumption with small effect sizes (0.09 to 0.28) over short (5 weeks or less) and long-term intervals (6 weeks or more) [11]. In a review in 2011 including 19 randomized controlled trial (RCT) studies where the student population represented the largest proportions of participants, a significant reduction in weekly alcohol consumption and binge drinking were found in student populations, but the findings were tentative because of methodological weaknesses in the studies [8]. In the most recent review in 2014 including 23 studies in which most were performed among student populations, online alcohol interventions were found effective in reducing consumption up to a 12-month period with a mean difference in consumption of approximately 1.5 to 2 standard drinks of alcohol [7].

After a large pilot study that successfully addressed study design issues [12-14], the AMADEUS-1 trial in Sweden [15-16] targeted both risky and nonrisky drinkers in a nontreatment-seeking student population and showed small but beneficial effects of assessment in comparison with a no-contact control group, with little additional impact of feedback [16]. AMADEUS-1 used an unconventional trial design with students unaware they were participating in a trial [15]. We preferred not to alter the intervention content of the national system based on this single evaluation study with an unconventional trial design. These considerations led us to design the subsequent study, AMADEUS-2, as a conventional 2-arm RCT design targeting hazardous and harmful drinkers only, using a single screening question and no baseline assessment to minimize assessment reactivity [4,17]. AMADEUS-2 [18] aims to provide a further evaluation of the national system for online alcohol intervention used in routine practice among the student health care centers in Sweden, specifically among the key target population of hazardous and harmful drinkers.

## Methods

### Study Design and Hypothesis

The study was a 2-arm parallel group RCT in which routine provision of single-session online alcohol assessment and feedback intervention (Group 1) was compared with nonintervention (Group 2) by delaying access to the intervention for 2 months until research follow-up was completed. Sweden is the first country to implement a national system of proactive alcohol intervention for students via student health care services. However, the timing of intervention delivery varies across Sweden and we took advantage of this lack of standardization of timing to implement random allocation at the individual level in this effectiveness evaluation study. The primary hypothesis was that the intervention group would reduce their total weekly alcohol consumption compared to the control group after 2 months. Ethical approval for the study was granted by the Regional Ethical Committee in Östergötland, Sweden (no: 2013/46-31).

### Study Procedures Including Recruitment and Randomization

The study was undertaken in 9 of 26 student health care centers in Sweden, each providing services to one university or college. These centers were selected on the basis that they had not previously been involved in RCTs in our research program. All students in their 2nd, 4th, or 6th terms (n=54,507) at the 9 colleges/universities were sent an email in March 2013 inviting them to answer a single question about their drinking. If eligible for trial participation, they were provided with information permitting fully informed consent, making this study unlike AMADEUS-1. The number of students invited varied across the colleges/universities according to their size from 831 in the smallest college (Gävle) to 13,102 in the largest university (Lund). The single screening question used was the third item of the Alcohol Use Disorders Identification Test (AUDIT) questionnaire on the frequency of heavy episodic drinking (HED) [19], used here with a 3-month timeframe. Single alcohol screening questions have been validated as identifying hazardous and harmful drinkers in different settings [20-21] and this type of drinking is particularly important in this population [22]. Students who were drinking 5 standard drinks (12 grams of alcohol in Sweden) or more for men or 4 standard drinks or more for women twice a month or more often were deemed eligible for trial participation. This approach was previously used by Walters and colleagues [23] who were similarly concerned to avoid reactivity to screening. The key underlying problem to be avoided was that assessment and intervention effects could interact to bias estimates of the effects of behavioral interventions in trials [15,16,24].

The initial email routinely sent from the participating student health care center was altered to invite study participation. Eligible consenting students were immediately randomized to intervention or control conditions. Randomization was done using Java’s built in random number generator (java.util.Random); thus, randomization was fully computerized, did not employ any strata or blocks, and all subsequent study processes were fully automated (programmed by MB). Unlike the AMADEUS-1 trial [14], there was no blinding in this study. The former group gained immediate access to the intervention and the latter group were informed that they would be able to access the intervention in 2 months’ time. Two months later, both groups were sent an identical email by the researchers. This email contained an invitation to participate in the follow-up survey, which included the same questions and type of feedback used in the baseline intervention received by the intervention group 2 months earlier. There were a total of 4 reminders (making 5 opportunities to respond in all), initially at weekly intervals, then at shorter intervals, with the final email making clear that this was the last opportunity to respond and allowing 2 days to do so. There were no incentives used to encourage study participation or retention.

### Intervention Content

**Figure 1:**
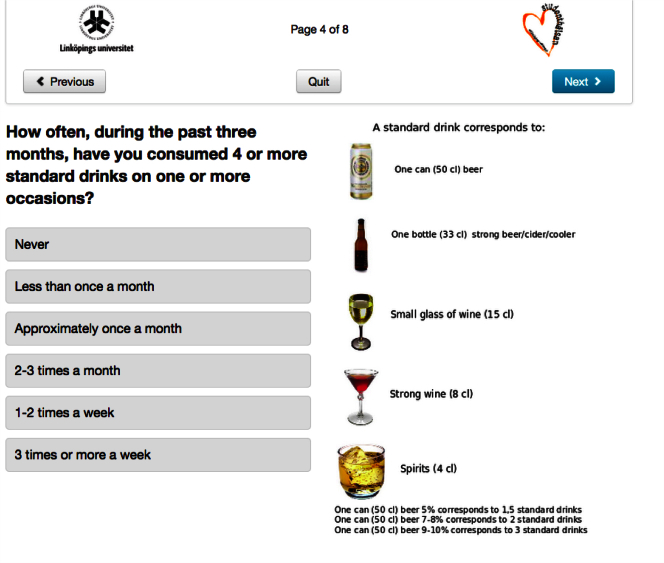
Screenshot of AMADEUS: assessment of heavy episodic drinking.

**Figure 2:**
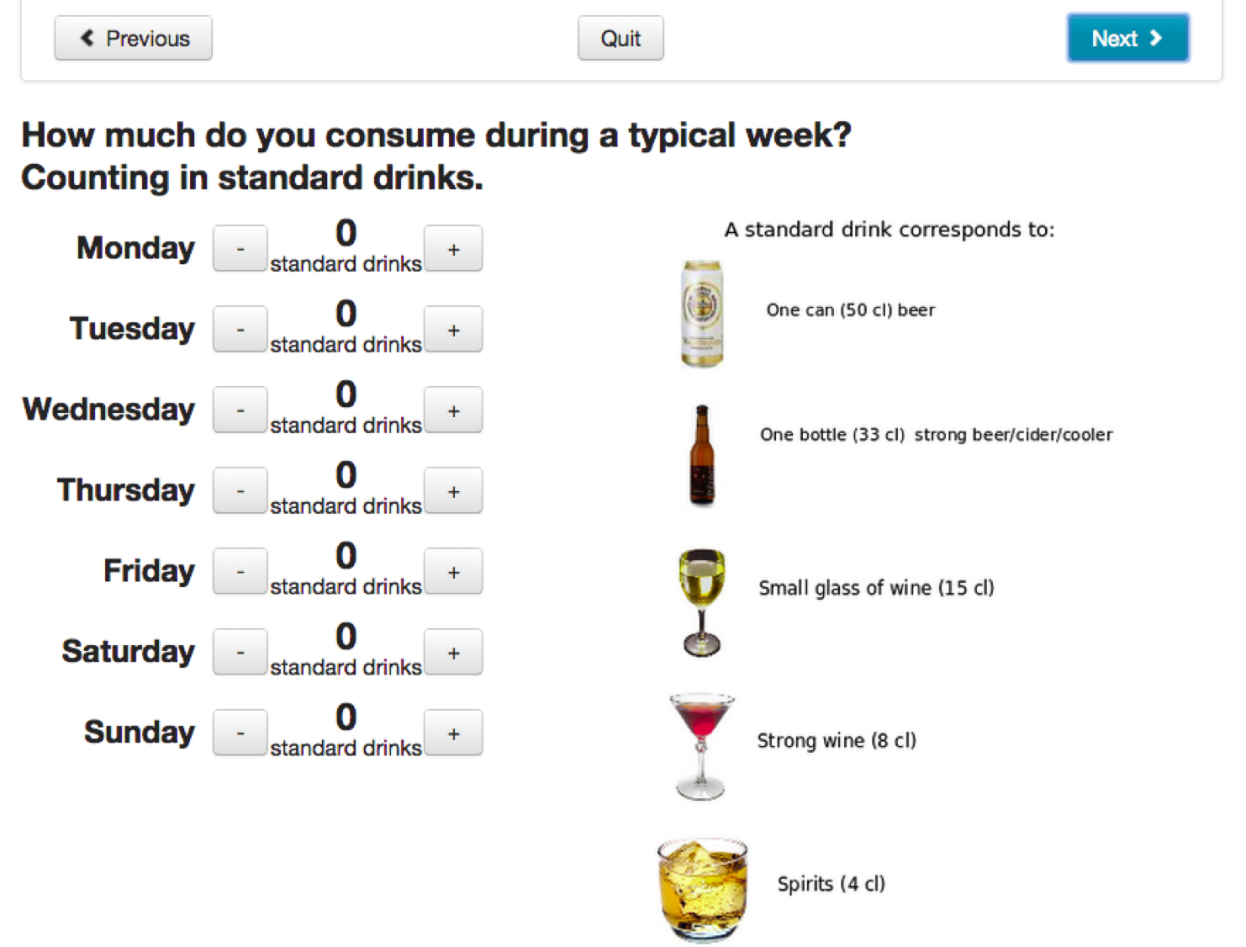
Screenshot of AMADEUS: assessment of weekly drinking.

**Figure 3:**
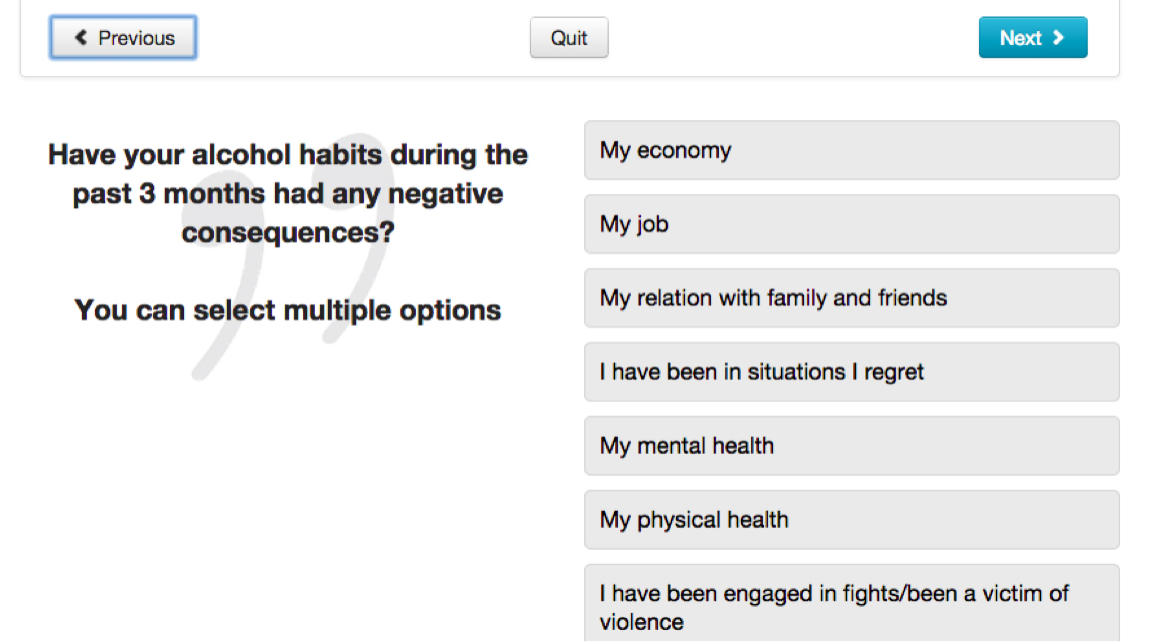
Screenshot of AMADEUS: assessment of negative consequences.

Immediately after randomization, the intervention group only were asked to complete an assessment. Four questions were asked about sex, age, domestic situation, and faculty of study. Alcohol consumption was calculated as total number of standard drinks for each of the 7 days in a typical week during the last 3 months (this intervention component was also later used as the primary outcome in the trial and note was unavailable for the control group to minimize reactivity); other questions explored frequency of HED, the largest amount of alcohol intake in standard drinks on a single occasion during the last 3 months, negative experiences perceived to be related to alcohol, and motivation to reduce alcohol consumption (Figures 1-3). Participants then received feedback consisting of 3 statements summarizing their weekly consumption, their frequency of HED, and their highest blood alcohol concentration during the last 4 weeks, comparing drinking patterns against the safe drinking limits established by the Swedish Institute for Public Health [25]. Also, a graphic illustration of their level of risk was given using green, yellow, and red colors to indicate risk status. After this followed comprehensive normative feedback with information describing participants’ alcohol use compared to their peers in Swedish universities (adjusted for sex and age group) and, if applicable, personalized advice on reducing unhealthy levels or patterns of consumption. The feedback was shown on the screen and could also be printed out by the student. A PDF version of the feedback was also emailed to the students immediately after closing this page. A demonstration version in English of the assessment and feedback intervention can be viewed online [26].

### Sample Size

The marginal costs involved in delivering online interventions to large numbers of participants in both routine practice and in scientific studies are low and much lower than other brief interventions after the developmental costs are met [21-22]. Therefore, even very small effects are likely also to be highly cost effective above the basic threshold cost involved in providing the service. These observations led us to believe that the sample should be as large as possible in order to detect very small effects. To assist study planning, we undertook an illustrative power calculation. To detect an effect size of *d*=0.1 standard deviations between the 2 groups with 5% significance level and 80% power required 1600 individuals analyzed per group. Assuming a follow-up rate of 50%, we aimed at recruiting 3200 individuals per group (ie 6400 in total). We had no data on the number of screen positives who might be willing to participate in the trial, but assumed approximately 70% would do so, meaning that we would need to identify approximately 8000 hazardous and harmful drinkers. In order to identify these number of participants, we needed to send emails to approximately 40,000 students with an average response rate of 40% (ie, n=16,000) and a 50% prevalence rate. We could not be confident of these estimates because, for example, patterns of email use vary considerably between colleges, being compulsory in some institutions and rarely used in others. Therefore, we decided to undertake the study in 9 colleges/universities with a total student enrollment of 54,507 students.

### Outcome Evaluation

This study used a single 2-month follow-up assessment interval, after which the control group gained access to the intervention. Thus, this study provides direct information only on the short-term effects of the intervention, although we stated a priori that if we found no short-term effects, we would not expect any longer-term effects [18].

The primary outcome was total weekly alcohol consumption. This was computed as the sum of alcohol consumed in standard drinks for each of the 7 days in a typical week, with data on each day of the week provided separately. Secondary outcomes were the proportions of students still drinking above national guidelines [25], frequency of drinking (number of days per week), quantity of drinks per drinking day, frequency of HED as defined in the screening question, highest estimated blood alcohol concentration (eBAC), and motivation to change.

Because there was no research assessment at study entry, information at this point was restricted to university, term, time from sending of invitation email to consent, and the frequency of HED from the screening question. At follow-up, we obtained additional information that was not possible, or not likely, to have been altered or altered differentially during the study period and which we used to examine equivalence: age, gender, weight, faculty of study, domestic status, and language used to answer the assessment and feedback language (Swedish or English). We also used measures of engagement with the study (device used, number of follow-up emails sent, and elapsed time before follow-up was completed).

### Statistical Methods

All outcomes were compared between randomized groups under the intention-to-treat principle (ie, including all randomized individuals in their originally randomized groups). The characteristics of responders at follow-up were compared between groups using chi-square tests or Fisher’s exact test for comparison of proportions and Student’s *t* test for comparison of means. Wilcoxon rank sum test was used to compare groups regarding time to follow-up. A linear trend test was applied to detect a possible trend in proportion of responses in relation to the number of email reminders before responding. Continuous outcome measures were assessed for skewness by visual inspection of histograms and Q-Q plots. Skewed continuous outcome were analyzed by negative binomial regression, and results are reported as percent reduction. Drinking above national guidelines was analyzed by logistic regression and reported as percentage reduction in odds. Frequency of HED occasions was analyzed by ordered logistic regression and reported as a percentage reduction in odds for exceeding any level. All regression analyses were performed first unadjusted and then adjusted for frequency of HED at baseline, age, university, and gender using the first 2 as continuous variables (thus allowing for dependence between individuals in the same university); the adjusted analysis was specified a priori as the primary result. A sensitivity analysis excluded 3 outliers in the follow-up assessment with extreme reported weekly consumption values.

Missing outcome data were initially handled by a complete-case analysis assuming that the data were missing at random (MAR). If data were not MAR, then nonresponders differed systematically from responders and early responders were likely to differ systematically from late responders, who were likely to be more similar to nonresponders [23]. Therefore, we explored the plausibility of the MAR assumption by regressing the primary outcome on the number of follow-up emails needed before an individual responded using a negative binomial regression in responders: a significant association would cast doubt on the MAR assumption. To allow for the possibility of data being missing not at random, we fitted the repeated attempts model of Jackson et al [27]. This model was not available in standard software for negative binomial regression, so we applied it to a linear regression of log (alcohol consumption + k), where k=24 units/week was chosen to eliminate skewness.

Tests for whether the intervention effect was modified by frequency of HED at baseline, age, university, and gender were undertaken for the primary outcome only and the first 2 were used as continuous variables. A post hoc sensitivity analysis accounted for possible heterogeneity between universities of treatment effects on weekly alcohol consumption using a 2-stage approach. The treatment effects on weekly alcohol consumption were first estimated in each university separately by negative binomial regression (adjusted for frequency of HED at baseline, age, and gender using the first 2 as continuous variables) and were then combined in a random effects meta-analysis.

## Results

### Study Population Characteristics

**Figure 4:**
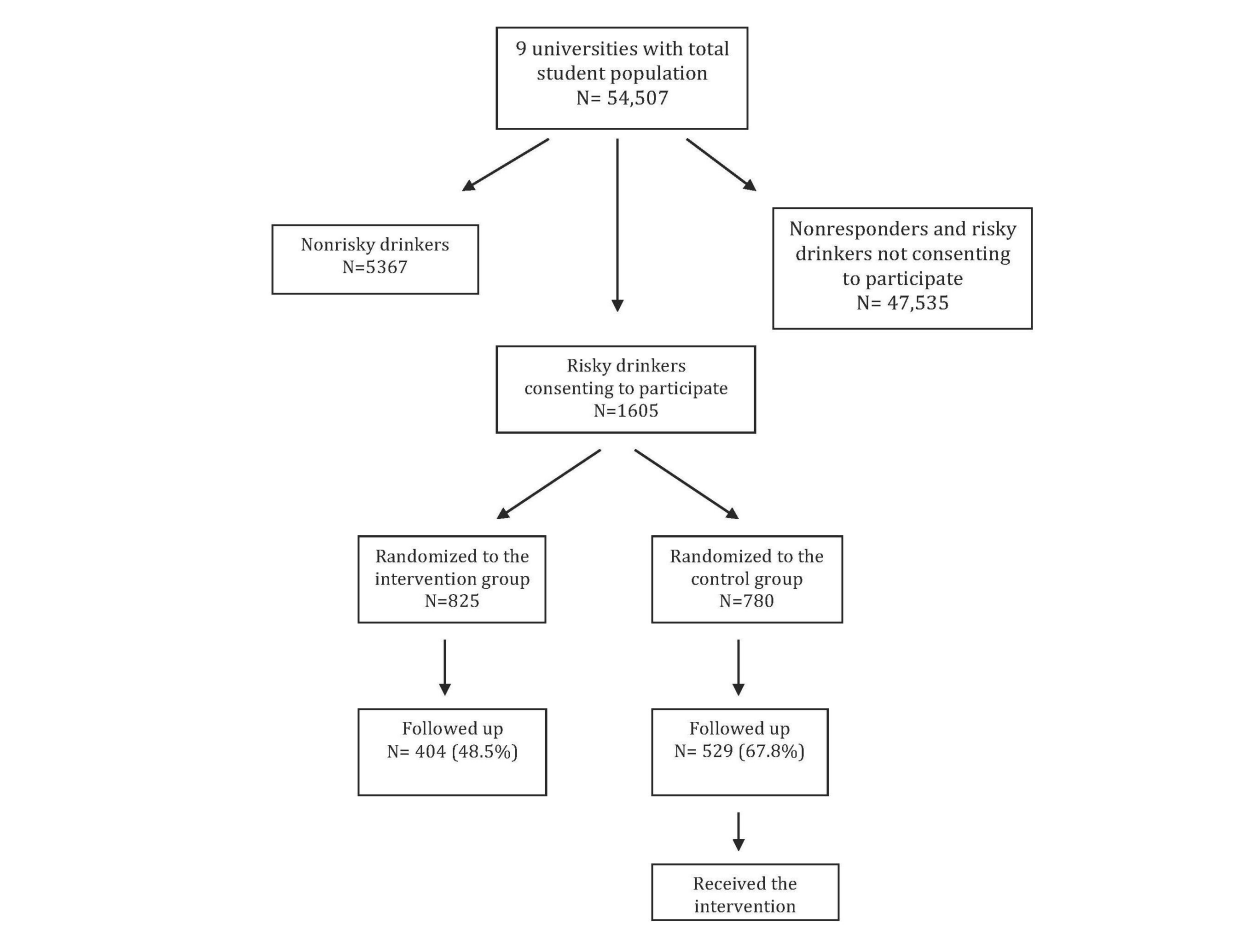
Flowchart of the AMADEUS study.

Figure 4 depicts the flow of participants from the invitation to the follow-up. In total, 1605 risky (both hazardous and harmful) drinkers agreed to participate in the study and were randomized to the intervention arm (n=825) or control arm (n=780). In Table 1, the intervention and control groups are compared at baseline for frequency of HED, term, and time to consent. Some of the smaller participating college/universities only managed to recruit a few risky drinkers. For one college (Gävle), none of the 4 recruited participants were randomized to the control group, so this college was necessarily excluded from the remaining analyses including college. Two large universities (Lund and Uppsala) contributed approximately two-thirds of all participants. There were no differences between the intervention and control groups with regard to baseline characteristics.

**Table 1:** Comparison of groups at baseline (N=1605).

Baseline data		Intervention (n=825)	Control (n=780)
**HED**^a^ **occasions, n (%)**			
	2-3 times a month	434 (52.6)	422 (54.1)
	1-2 times a week	350 (42.4)	323 (41.4)
	≥3 times a week	41 (5.0)	35 (4.5)
**University, n (%)**			
	Blekinge	23 (2.8)	31 (4.0)
	Linné	85 (10.3)	65 (8.3)
	Malmö	78 (9.5)	57 (7.3)
	Lund	293 (35.5)	275 (35.3)
	Gävle	4 (0.5)	0 (0.0)
	Halmstad	27 (3.3)	38 (4.9)
	Mälardalen	21 (2.5)	27 (3.5)
	Skövde	24 (2.9)	22 (2.8)
	Uppsala	270 (32.7)	265 (34.0)
**Term, n (%)**			
	2	352 (42.7)	306 (39.2)
	4	263 (31.9)	251 (32.2)
	6	210 (25.5)	223 (28.6)
Time to consent (hours), median (IQR)		93 (4-200)	102 (5-190)

^a^ HED: Heavy episodic drinking assessed by the question “How often, during the past 3 months, have you consumed 4 (women) or 5 (men) standard drinks on 1 occasion?”

The control group were much more likely to participate at follow-up (67.8% vs 49.0%, *P*<.001). Table 2 compares the characteristics of these responders in the intervention and control group at follow-up. None of the characteristics unlikely to have been altered since baseline differed between the 2 groups. There was a statistically significant decrease in the proportion of responses in relation to the number of email reminders before responding (*P*=.02).

**Table 2:** Comparison of groups at follow-up^a^ (N=931).

Characteristics			Intervention (n=402)	Control (n=529)	*P*^b^
**Characteristics unlikely to have changed since baseline**					
	**Gender, n (%)**^c^				
		Male	198 (49.3)	276 (52.2)	.38
		Female	204 (50.7)	253 (47.8)	
	**Age (years), n (%)**^c^				
		<18	62 (15.4)	81 (15.3)	.60
		18-20	271 (67.4)	366 (69.2)	
		21-25	49 (12.2)	65 (12.3)	
		26-30	20 (5.0)	17 (3.2)	
	**Faculty of study, n (%)**^c^				
		Science and engineering	128 (31.8)	168 (31.8)	.90
		Humanities	213 (53.0)	286 (54.1)	
		Medical	61 (15.2)	75 (14.2)	
	**Language used, n (%)**^c^				
		Swedish	387 (96.3)	511 (96.6)	.79
		English	15 (3.7)	18 (3.4)	
	**Domestic status, n (%)**				
		Living alone without kids at home	267 (66.4)	360 (68.1)	.06
		Living alone with kids at home	3 (0.7)	1 (0.2)	
		Living with somebody without kids	74 (18.4)	101 (19.1)	
		Living with somebody with kids	15 (3.7)	6 (1.1)	
		Have a partner but not living together	43 (10.7)	61 (11.5)	
	**Domestic status (3 categories), n (%)**				
		Living alone	270 (67.2)	361 (68.2)	.75
		Living with somebody	89 (22.1)	107 (20.2)	
		Have a partner but not living together	43 (10.7)	61 (11.5)	
	Weight (kg), mean (SD)^d^		71.53 (13.35)	70.92 (12.24)	
**Characteristics specific to follow-up**					
	**Device used, n (%)**				
		Mobile phone	85 (21.1)	129 (24.4)	.39
		Laptop	302 (75.1)	376 (71.1)	
		Tablet	15 (3.7)	24 (4.5)	
	Time to follow-up (hours), median (IQR)		71 (279)	48 (207)	0.31
	**Number of follow-up emails before response, n (%)**				
		1	222 (55.2)	315 (59.6)	.07^e^
		2	82 (20.4)	103 (19.5)	
		3	51 (12.7)	70 (13.2)	
		4	27 (6.7)	23 (4.3)	
		5	20 (5.0)	18 (3.4)	

^a^ Without university of Gävle; this is the population used for the primary analyses in Table 3.

^b^ All *P*-values were provided for heterogeneity except for variable number of follow-up emails. Determined using chi-square test (gender, age, faculty, language, domestic status 3 categories), Fisher exact test (domestic status), Student’s *t* test (weight), Wilcoxon rank sum test (time to follow-up), or linear trend test (number of follow-up emails).

^c^ Regarded as baseline variables in the analysis.

^d^ Intervention: n=401; control: n=528.

^e^ Trend test.

### Main Findings

The main outcomes are displayed in Table 3. Small differences indicative of lower risk consumption were observed for the intervention group for all primary and secondary outcomes. However, none of these differences were statistically significant in the primary adjusted analyses.

**Table 3:** Trial outcomes (n=931).

Outcomes			Intervention (n=402)	Control (n=529)	% Reduction in mean or odds^a^			
					Unadjusted		Adjusted^b^	
					% (95% CI)	*P*	% (95% CI)	*P*
**Primary outcome**								
	Weekly alcohol consumption (g/week), mean (SD)		113.4 (81.1)	120.8 (86.4)	6% (–3%, 14%)	.18	6% (–2%, 13%)	.13
**Secondary outcomes**								
	Proportion drinking above national guidelines,^c^ n (%)		370 (92.0)	492 (93.0)	13% (–42%, 47%)	.58	5% (–59%, 43%)	.85
	Frequency of drinking (days/week), mean (SD)		2.30 (1.52)	2.34 (1.53)	2% (–7%, 10%)	.67	1% (–8%, 9%)	.85
	Number of drinks per drinking day,^d^ mean (SD)		4.5 (2.6)	4.7 (2.7)	5% (–2%, 12%)	.14	4% (–3%, 11%)*P*	.23
	**Frequency of HED occasions, n (%)**				16% (–7%, 33%)	.17	14% (–10%, 33%)	.24
		Never	11 (2.7)	8 (1.5)				
		<1 time a month	22 (5.5)	29 (5.5)				
		Approximately once a month	64 (15.9)	82 (15.5)				
		2-3 times a month	169 (42.0)	207 (39.1)				
		1-2 times a week	128 (31.8)	187 (35.3)				
		≥3 times a week	8 (2.0)	16 (3.0)				
	Highest eBAC, mean (SD)		1.16 (1.08)	1.31 (1.14)	11% (0%, 21%)	.05	11% (–1%, 20%)	.06
	**Motivation to change,** ^e^ **n (%)**				4% (–21%, 25%)	.71	2% (–24%, 23%)	.86
		I have had no thoughts about decreasing	175 (43.6)	225 (42.6)				
		I have thought about decreasing, but I am not thinking about it right now	87 (21.7)	108 (20.5)				
		I am thinking about how I will decrease	28 (7.0)	50 (9.5)				
		I have started decreasing	105 (26.2)	135 (25.6)				
		I have tried to decrease, but failed	6 (1.5)	10 (1.9)				

^a^ Of intervention compared to control. Reduction in mean by negative binomial regression: weekly alcohol consumption, frequency of drinking, number of drinks per drinking day, and highest eBAC; reduction in odds by logistic regression: proportion drinking about national guidelines; reduction in odds of exceeding any cutoff by ordered logistic regression: frequency of HED occasions, motivation to change.

^b^ Adjusted for frequency of heavy episodic drinking at baseline, age, university, and gender, using the first 2 as continuous variables.

^c^ Risky drinker: heavy episodic drinking (HED) >once per month and/or total weekly consumption >14 standard drinks (men) or 9 (women).

^d^ Intervention: n=395; control: n=523.

^e^ Intervention: n=401; control: n=528.

### Additional Analyses

**Figure 5:**
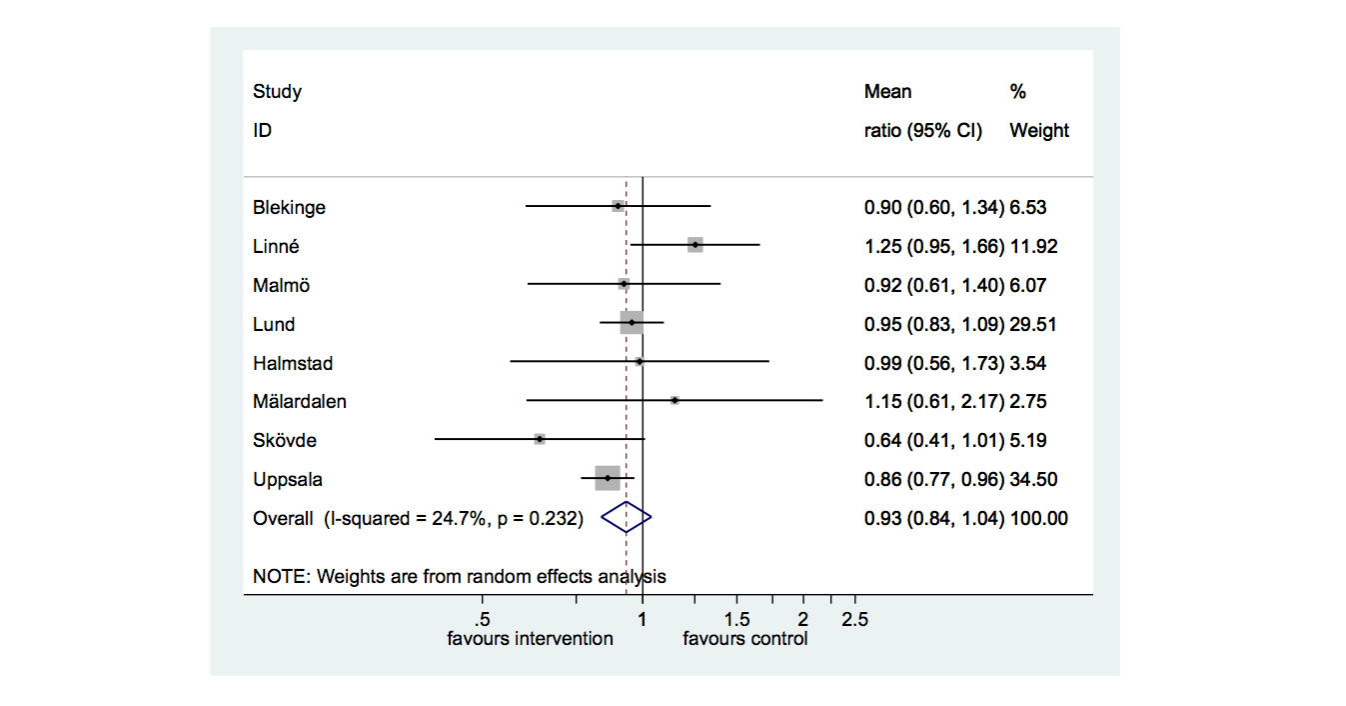
Forest plot of ratio of means in weekly alcohol consumption comparing intervention to control.

Effect modification analyses did not reveal any statistically significant findings. However, the interaction between university and randomized group had a *P*-value of .07. Therefore, we explored this in a post hoc sensitivity analysis allowing the treatment effect to vary by university. The random effects meta-analysis (Figure 5) showed a 7% reduction in weekly alcohol consumption that was not statistically significant (95% CI –16% to 4%, *P*=.20). The confidence intervals of the analysis adjusted by cluster were somewhat wider than in the unadjusted analysis.

We also considered the statistically significant between-group difference for Uppsala (n=365), where weekly alcohol consumption was approximately 14% lower (95% CI –23% to –4%, *P*=.009 adjusted) in the intervention group than the control group. Further analysis of 3 secondary outcomes for Uppsala University also showed a significant difference in number of drinks per drinking day with 13% fewer (95% CI –23% to 3%, *P*=.01 adjusted), but no significant differences for frequency of drinking and highest eBAC.

In the assessment for skewness of the continuous variables, we found 1 outlier in the treatment group (with weekly alcohol consumption of 1044 g/week) and 2 outliers in the control group (with weekly alcohol consumption of 1128 and 1524 g/week). The maximum reported weekly alcohol consumption of those not excluded was 552 g/week in the treatment group and 456 g/week in the control group. Therefore, we performed a sensitivity analysis without these outliers. In this analysis, which was not specified a priori, the between-group difference in the primary outcome, weekly alcohol consumption, in the primary adjusted analysis, crossed the conventional threshold for statistical significance (8% reduction, 95% CI –15% to 0%, *P*=.049 adjusted; 10% reduction, 95% CI –17% to –1%, *P*=.02 unadjusted). No statistically significant differences were seen for the secondary outcomes in the primary adjusted analyses, although eBAC was statistically significant in the unadjusted analysis (11% reduction, 95% CI 0% to 21%, *P*=.047) but did not meet significance in the adjusted analysis (11% reduction, 95% CI –21% to 1%, *P*=.06).

The preceding analyses assumed the data were MAR. There was no statistically significant association between the primary outcome and the number of email reminders before answering the follow-up (*P*=.71), so the data are consistent with the MAR assumption. Another post hoc analysis assessed time to consent and found no association and thus no evidence that data were not MAR. Analyses using the repeated attempts model and linear regression suggested an intervention effect of a 10% reduction (95% CI –30% to 10%).

## Discussion

The study found no strong evidence of short-term effectiveness of the Swedish national system of proactive online alcohol intervention for university and college students. However, inspection of the confidence intervals for the primary outcome (in Table 3) reveals that this study does not rule out an intervention effect of up to 13% reduction in total weekly alcohol consumption. The sensitivity analysis excluding outliers suggests an intervention effect on reduced total weekly alcohol consumption, although the statistical significance attained in that analysis should be treated with caution because that particular analysis was not prespecified. We have no reason to anticipate later occurring effects because brief intervention effects generally wane with time [5] and the short-term nature of this evaluation study is important to note in interpreting study findings. The best estimate of the intervention effect in Uppsala is larger (14% reduction in total weekly alcohol consumption compared to 6% across Sweden as a whole) and is statistically significant. This finding should be interpreted as hypothesis generating because, although prespecified, the differences in intervention effect across the universities as a whole were not statistically significant.

We did not have statistical power to detect the effect size that we believed was worth obtaining in the planning of this study and this is a clear study limitation. We succeeded in recruiting only one-quarter of our target sample size and the best estimate of the effect obtained on the primary outcome (a 6% reduction in alcohol consumption) is of clear public health significance. For example, it is very close to the size of effects considered appropriate for the implementation of face-to-face brief intervention programs [28] and measures to increase the price of alcohol [29]. Although we set out to evaluate a national system, we only managed to recruit 3% of all individuals invited, although many of those who did not respond will have simply ignored the email invitation as not relevant to them (eg, because they did not drink much or at all).

The contrast with AMADEUS-1 in rates of participation is very striking. Conventional trial recruitment in AMADEUS-2 resulted in less than 1000 hazardous and harmful drinkers providing follow-up data from more than 54,000 initial invitations to consider participation in 9 universities and colleges. In AMADEUS-1, more than 1500 hazardous and harmful drinkers in 2 of the 3 arms provided follow-up data after completing baseline assessments, among approximately 7800 providing follow-up data from less than 15,000 targeted for study in 2 universities. Even allowing for differences in use of email between universities, the conventional study design with informed consent clearly impacts on participation in detrimental ways. However, other differences between the studies should be borne in mind. AMADEUS-2 provides an intention-to-treat evaluation of effects among hazardous and harmful drinkers, whereas a per-protocol analysis only was possible in AMADEUS-1 for this group due to the nature of the study design. Participation rates in AMADEUS-2 nevertheless expose the limitations of unblinded conventional trials designed to detect small effects of public health significance; it is not possible to undertake a fully powered study among student risky drinkers in Sweden, a country of approximately 10 million people. The external validity of the current findings, bearing in mind the low participation rate, warrants careful consideration.

Reliably detecting small effects is challenging and subject to the play of chance, and likely also to be influenced by a number of contextual factors that are difficult to capture. For example, the timing of follow-up within the academic year might be relevant because campus activities involving alcohol may both vary and influence study findings. Initial follow-up in this study was undertaken as exams approached.

The extent of differential attrition provides additional reasons for avoiding strong conclusions because the potential for selection bias exists, even though our analysis revealed no evidence to contradict our MAR assumption in relation to the missing data. It is also important to note that this intervention is not designed to meet the needs of problem drinkers [30] and online interventions extending over several sessions or contacts and/or person-to-person interactions are likely to be needed.

Previous online alcohol studies among college and university students have shown mixed results [6,7,29]. Apart from AMADEUS-1, there have been few randomized studies capable of dismantling components of effective interventions in this or similar populations. The content of this Swedish intervention is broadly similar to the THRIVE intervention evaluated in one university in Australia [31] and the New Zealand e-SBINZ trials intervention in comprising normative feedback, criterion feedback (on guidelines), and brief advice [32]. The effects are also broadly similar, for example with THRIVE showing a 17% reduction in alcohol consumption in comparison with controls after 1 month and decreasing to 11% after 6 months [31]. Some effects among Maoris were somewhat larger, although effects among non-Maori in the parallel e-SBINZ trial were smaller [32,33]. AMADEUS-1 [16] found that feedback added little to the effects of assessment and detailed investigations of intervention content are urgently needed in order to ascertain whether it may be possible to develop novel interventions or intervention components capable of larger effects than have been identified to date. This underdevelopment of online study is similar to that which pertains for face-to-face brief interventions [34].

The study has attempted to evaluate the effectiveness of an existing national service provision across a sizable number of institutions. Although it does not provide strong evidence of benefit, it is unclear how far the existing intervention should be redesigned, if at all, on the basis of these findings and those of AMADEUS-1. Further research and development work is needed, particularly in light of the low costs involved and the consequent likelihood of high levels of cost-effectiveness being associated with even very small effects. The key research challenge is to robustly identify and control for biases that interfere with reliable estimation of small effects [24,25,27-35]. Randomized controlled trials that directly compare the performance of the existing online intervention with novel candidates to augment or replace it are appropriate in this situation. Further investment in such work should not detract from the need for other population-level alcohol interventions, such as increasing price, better controlling availability, and restricting marketing to change the cultural acceptability of heavy drinking. The existing evidence suggests that these types of alcohol policies are most likely to be effective and we do not know whether or how far individual-level interventions in whole populations such as that evaluated here may enhance the anticipated effects [36].

## Appendix

Multimedia Appendix 1 CONSORT-EHEALTH Checklist V 1.6.2 [37].

## Abbreviations

AUDIT: Alcohol Use Disorders Identification Test
eBAC: estimated blood alcohol concentration
HED: heavy episodic drinking
MAR: missing at random
RCT: randomized controlled trial
