# Correction: Online Alcohol Assessment and Feedback for Hazardous and Harmful Drinkers: Findings From the AMADEUS-2 Randomized Controlled Trial of Routine Practice in Swedish Universities

**DOI:** 10.2196/jmir.5724

**Published:** 2016-05-16

**Authors:** Preben Bendtsen, Marcus Bendtsen, Nadine Karlsson, Ian R White, Jim McCambridge

**Affiliations:** ^1^Department of Medical and Health SciencesLinköping UniversityLinköpingSweden; ^2^Department of Computer ScienceUniversity of LinköpingLinköpingSweden; ^3^Cambridge Institute of Public HealthMRC Biostatistics UnitCambridgeUnited Kingdom; ^4^Faculty of ScienceDepartment of health SciencesUniversity of YorkHeslingtonUnited Kingdom

The authors of the paper “Online Alcohol Assessment and Feedback for Hazardous and Harmful Drinkers: Findings From the AMADEUS-2 Randomized Controlled Trial of Routine Practice in Swedish Universities” (J Med Internet Res 2015;17(7):e170) used the wrong variable for the number of email reminders before participants answered the follow-up. In Table 2, the values in columns two, three, and four under the subcategory "Number of follow-up emails before response, n (%)" should read as in the table below. A footnote has also been added to the *P*-value associated with this variable. In the Results section, there remains no statistically significant association between the primary outcome and the number of email reminders (*P*=.16), consistent with the missing at random (MAR) assumption. However, analyses using the repeated attempts model and linear regression suggested an intervention effect of an 11% reduction (18% reduction to 3% reduction) and a statistically significant departure from the MAR assumption. The authors are unable to explain why this result contradicts the above analysis that uses the same variables, so they prefer to trust the simpler model and hence they do not regard the repeated attempts model findings as providing any strong evidence against the MAR assumption. This error has been corrected in the online version of the paper on the JMIR website on May 16, 2016 together with publishing this correction notice. A correction notice has been sent to PubMed and PubMed Central.

**Table 2:** Comparison of groups at follow-up (total n=931 without University of Gävle).

Number of follow-up emails before response, n (%)	Intervention (n=402)	Control (n=529)	*P*-value
1	222 (55.2)	315 (59.6)	.07 ^a^
2	82 (20.4)	103 (19.5)	
3	51 (12.7)	70 (13.2)	
4	27 (6.7)	23 (4.3)	
5	20 (5.0)	18 (3.4)	

^a^Trend test.

