# Supplementary material for: Online Alcohol Assessment and Feedback for Hazardous and Harmful Drinkers: Findings From the AMADEUS-2 Randomized Controlled Trial of Routine Practice in Swedish Universities
Source: J Med Internet Res. 2015 Jul 9;17(7):e170. doi: 10.2196/jmir.4020 (PMC4526986; doi:10.2196/jmir.4020)
Supplement: Multimedia Appendix 1 [file jmir_v17i7e170_app1.pdf]

CONSORT-EHEALTH Checklist V1.6.2 Report

(based on CONSORT-EHEALTH V1.6), available at [http://tinyurl.com/consort-ehealth-v1-6].

4020

Date completed

3/25/2015 11:57:46

by

Preben Bendtsen

Online alcohol assessment & feedback for hazardous and harmful drinkers: findings from the AMADEUS--2 randomised controlled trial of routine practice in Swedish universities.

TITLE

1a-i) Identify the mode of delivery in the title

like this: "brief online alcohol intervention "

1a-ii) Non-web-based components or important co-interventions in title

We have no non-web-based components in our study

1a-iii) Primary condition or target group in the title

Like this: "online alcohol intervention used in routine practice among the student health care centres in Sweden, specifically among the key target population of hazardous and harmful drinker"

ABSTRACT

1b-i) Key features/functionalities/components of the intervention and comparator in the METHODS section of the ABSTRACT

Like this: "This study evaluates the effectiveness of national provision of a brief online alcohol intervention for students in Sweden.
Risky drinkers (n=1,605) at 9 colleges and universities in Sweden were identified using a single screening question. These students gave consent and were randomised into a two-arm parallel group RCT, consisting of immediate or delayed access to the intervention."

1b-ii) Level of human involvement in the METHODS section of the ABSTRACT

Like this: "a fully automated online assessment and intervention with personalized feedback".

1b-iii) Open vs. closed, web-based (self-assessment) vs. face-to-face assessments in the METHODS section of the ABSTRACT

Like this: "Risky drinkers (n=1,605) at 9 colleges and universities in Sweden were invited by mail and identified using a single screening question"

1b-iv) RESULTS section in abstract must contain use data

Like this: Although there were some indications of possible benefit in sensitivity analyses, suggesting an intervention effect of a 10% reduction (95% CI 10 % increase to 30 % reduction)

1b-v) CONCLUSIONS/DISCUSSION in abstract for negative trials

Like this: "Any effects of current national provision are likely to be small and further research and development work is needed to enhance effectiveness

INTRODUCTION

2a-i) Problem and the type of system/solution

Like this: "Existing evidence of the effectiveness of online interventions with students is mixed [6-8], partly because of unresolved methodological challenges including attrition prevention and assessment reactivity"

2a-ii) Scientific background, rationale: What is known about the (type of) system

Like this: "Existing evidence of the effectiveness of online interventions with students is mixed [6-8], partly because of unresolved methodological challenges including attrition prevention and assessment reactivity"

METHODS

3a) CONSORT: Description of trial design (such as parallel, factorial) including allocation ratio

Like this: "AMADEUS-2 aims to provide a further evaluation of the national system for online alcohol intervention used in routine practice among the student health care centres in Sweden, specifically among the key target population of hazardous and harmful drinkers."

3b) CONSORT: Important changes to methods after trial commencement (such as eligibility criteria), with reasons

No changes was done

3b-i) Bug fixes, Downtimes, Content Changes

No change was made

4a) CONSORT: Eligibility criteria for participants

Like thia " Students who were drinking twice a month or more often, 5 standard drinks (12 grams of alcohol in Sweden) or more for men or 4 standard drinks or more for women, were deemed eligible for trial participation. "

4a-i) Computer / Internet literacy

No specified in this trials. Alla participants were young students who were thought to be use to computers.

4a-ii) Open vs. closed, web-based vs. face-to-face assessments:

Like this: "All students in their 2nd, 4th or 6th terms (n=54,507) at the 9 colleges/universities were sent an e-mail in March 2013 inviting them to answer a single question about their drinking. If eligible for trial participation, they were provided with information permitting informed consent, making the present study unlike AMADEUS--1. "

4a-iii) Information giving during recruitment

Like this: " All students in their 2nd, 4th or 6th terms (n=54,507) at the 9 colleges/universities were sent an e-mail in March 2013 inviting them to answer a single question about their drinking. If eligible for trial participation, they were provided with information permitting informed consent, making the present study unlike AMADEUS--1."

4b) CONSORT: Settings and locations where the data were collected

Like this: "The study was undertaken in 9 of 26 student health care centres in Sweden, each providing services to one university or college. These centres were selected on the basis that they had not previously been involved in randomised controlled trials in our research programme"

4b-i) Report if outcomes were (self-)assessed through online questionnaires

Like this: This study used a single 2-month follow-up assessment interval, after which the control group gained access to the intervention"

4b-ii) Report how institutional affiliations are displayed

Like this: " The initial e-mail routinely sent from the participating student health care centre was altered so as to invite study participation"

5) CONSORT: Describe the interventions for each group with sufficient details to allow replication, including how and when they were actually administered

5-i) Mention names, credential, affiliations of the developers, sponsors, and owners

Like this: followed comprehensive normative feedback with information describing participants' alcohol use compared to their peers in Swedish universities (adjusted for sex and age group), and, if applicable, personalized advice on reducing unhealthy levels or patterns of consumption. The feedback was shown on the screen and could also be printed out by the student. A pdf version of the feedback was also e-mailed to the students immediately after closing this page. A demonstration version in English of the assessment and feedback intervention can be viewed at http://demo.livsstilstest.nu."

5-ii) Describe the history/development process
Like this: " AMADEUS-1 used an unconventional trial design with students unaware they were participating in a trial [16]. We preferred not to alter the intervention content of the national system on the basis of a single evaluation study with an unconventional trial design. These considerations led us to design the subsequent study, AMADEUS-2, as a conventional 2 arm randomised controlled trial (RCT) design targeting hazardous and harmful drinkers only, using a single screening question and no baseline assessment in order to minimise assessment reactivity [4,15]. "

5-iii) Revisions and updating

No change was done

5-iv) Quality assurance methods

No specific checked. The follow--up was done by online self-assessment.

5-v) Ensure replicability by publishing the source code, and/or providing screenshots/screen-capture video, and/or providing flowcharts of the algorithms used

Like this: A demonstration version in English of the assessment and feedback intervention can be viewed at http://demo.livsstilstest.nu."

5-vi) Digital preservation

Like this: "A demonstration version in English of the assessment and feedback intervention can be viewed at http://demo.livsstilstest.nu."

5-vii) Access

The participants accessed the intervention on their own computer/tablett/mobile phone.

5-viii) Mode of delivery, features/functionalities/components of the intervention and comparator, and the theoretical framework
Like this: comprehensive normative feedback with information describing participants' alcohol use compared to their peers in Swedish universities (adjusted for sex and age group), and, if applicable, personalized advice on reducing unhealthy levels or patterns of consumption. The feedback was shown on the screen and could also be printed out by the student. A pdf version of the feedback was also e- mailed to the students immediately after closing this page. A demonstration version in English of the assessment and feedback intervention can be viewed at http://demo.livsstilstest.nu."

5-ix) Describe use parameters
Like this: " The study is a two-arm parallel group RCT in which routine provision of single-session online alcohol assessment and feedback intervention (Group 1) compared with non-intervention (Group 2), by delaying access to intervention for two months until research follow-up is completed. "

5-x) Clarify the level of human involvement

Fully automated system

5-xi) Report any prompts/reminders used

This was a single-session interventions with no reminders.

5-xii) Describe any co-interventions (incl. training/support)

NA

6a) CONSORT: Completely defined pre-specified primary and secondary outcome measures, including how and when they were assessed
Like this:" The primary outcome was total weekly alcohol consumption. This was computed as the sum of alcohol consumed in standard drinks for each of the 7 days in a typical week, with data on each day of the week separately provided. Secondary outcomes were the proportions of students still drinking above national guidelines [22], frequency of drinking (number of days per week), quantity of drinks per drinking day, frequency of HED as defined in the screening question, highest estimated blood alcohol concentration (eBAC) and stage of change."

6a-i) Online questionnaires: describe if they were validated for online use and apply CHERRIES items to describe how the questionnaires were designed/deployed

The questions used is similar to the validated AUDIT questionnaire commonly used in alcohol research

6a-ii) Describe whether and how “use” (including intensity of use/dosage) was defined/measured/monitored

Since this was a single session we did/could no measure how long time the individual spend on reading the feed-back.

6a-iii) Describe whether, how, and when qualitative feedback from participants was obtained

No qualitative feedback was obtained in this study

6b) CONSORT: Any changes to trial outcomes after the trial commenced, with reasons

NA

7a) CONSORT: How sample size was determined

7a-i) Describe whether and how expected attrition was taken into account when calculating the sample size
To detect an effect size d of 0.1 standard deviations between the two groups with 5% significance level and 80% power required 1,600 individuals analysed per group. Assuming a follow-up rate of 50%, we aimed at recruiting 3,200 individuals per group i.e. 6,400 in total. We had no data on the number of screen positives who might be willing to participate in the trial but assumed approximately 70% would do so, meaning that we would need to identify approximately 8,000 hazardous and harmful drinkers. In order to identify these number of participants, we needed to send e-mails to approximately 40,000 students with an average response rate of 40% (i.e. n=16,000) and a 50% prevalence rate. We could not be confident of these estimates as, for example, patterns of e-mail use vary considerably between colleges, being compulsory in some institutions and rarely used in others. We therefore decided to undertake the study in 9 colleges/universities with a total student enrolment of 54,507 students.

7b) CONSORT: When applicable, explanation of any interim analyses and stopping guidelines

NA

8a) CONSORT: Method used to generate the random allocation sequence
Like this: "Randomisation was done using Java’s built in random number generator (java.util.Random). Randomisation was thus fully computerized, did not employ any strata or blocks, and was not possible to subvert, as this and all subsequent study processes were fully automated (programmed by MB). "

8b) CONSORT: Type of randomisation; details of any restriction (such as blocking and block size)
Like this: Randomisation was done using Java’s built in random number generator (java.util.Random). Randomisation was thus fully computerized, did not employ any strata or blocks, and was not possible to subvert, as this and all subsequent study processes were fully automated (programmed by MB).

9) CONSORT: Mechanism used to implement the random allocation sequence (such as sequentially numbered containers), describing any steps taken to conceal the sequence until interventions were assigned
Like this: Randomisation was done using Java’s built in random number generator (java.util.Random). Randomisation was thus fully computerized, did not employ any strata or blocks, and was not possible to subvert, as this and all subsequent study processes were fully automated (programmed by MB).

10) CONSORT: Who generated the random allocation sequence, who enrolled participants, and who assigned participants to interventions
Like this: Randomisation was done using Java’s built in random number generator (java.util.Random). Randomisation was thus fully computerized, did not employ any strata or blocks, and was not possible to subvert, as this and all subsequent study processes were fully automated (programmed by MB).

11a) CONSORT: Blinding - If done, who was blinded after assignment to interventions (for example, participants, care providers, those assessing outcomes) and how

11a-i) Specify who was blinded, and who wasn’t

No blinding in this study

11a-ii) Discuss e.g., whether participants knew which intervention was the "intervention of interest" and which one was the "comparator"

No blinding

11b) CONSORT: If relevant, description of the similarity of interventions

NA

12a) CONSORT: Statistical methods used to compare groups for primary and secondary outcomes

All regression analyses were performed first unadjusted and then adjusted for frequency of heavy episodic drinking at baseline, age, university, and gender, using the first two as continuous variables (thus allowing for dependence between individuals in the same university); the adjusted analysis was specified a priori as the primary result.

Tests for whether the intervention effect was modified by frequency of HED at baseline, age, university, and gender were undertaken for the primary outcome only, using the first two as continuous variables. A post hoc sensitivity analysis accounted for possible hetereogeneity between universities of treatment effects on weekly alcohol consumption using a two-stage approach. The treatment effects on weekly alcohol consumption were first estimated in each university separately by negative binomial regression (adjusted for frequency of heavy episodic drinking at baseline, age, and gender, using the first two as continuous variables) and were then combined in a random-effects meta-analysis.

12a-i) Imputation techniques to deal with attrition / missing values

Missing outcome data were initially handled by a complete-cases analysis, assuming that the data were missing at random (MAR). If data are missing not at random then non-responders differ systematically from responders, and early responders are likely to differ systematically from late responders, who are likely to be more similar to non-responders [23]. We therefore explored the plausibility of the MAR assumption by regressing the primary outcome on the number of follow-up emails needed before an individual responded, using a negative binomial regression in responders: a significant association would cast doubt on the MAR assumption. To allow for the possibility of data being missing not at random, we fitted the repeated attempts model of Jackson et al. [26]. This model is not available in standard software for negative binomial regression, so we applied it to a linear regression of log (alcohol consumption + k), where k=24 units/week was chosen to eliminate skewness.

12b) CONSORT: Methods for additional analyses, such as subgroup analyses and adjusted analyses

Tests for whether the intervention effect was modified by frequency of HED at baseline, age, university, and gender were undertaken for the primary outcome only, using the first two as continuous variables. A post hoc sensitivity analysis accounted for possible hetereogeneity between universities of treatment effects on weekly alcohol consumption using a two-stage approach. The treatment effects on weekly alcohol consumption were first estimated in each university separately by negative binomial regression (adjusted for frequency of heavy episodic drinking at baseline, age, and gender, using the first two as continuous variables) and were then combined in a random-effects meta-analysis.

RESULTS

13a) CONSORT: For each group, the numbers of participants who were randomly assigned, received intended treatment, and were analysed for the primary outcome

Seen in figure 1

13b) CONSORT: For each group, losses and exclusions after randomisation, together with reasons

Seen in figure 1

13b-i) Attrition diagram

NA

14a) CONSORT: Dates defining the periods of recruitment and follow-up

No

14a-i) Indicate if critical "secular events" fell into the study period

NA

14b) CONSORT: Why the trial ended or was stopped (early)

NA

15) CONSORT: A table showing baseline demographic and clinical characteristics for each group

Tabel 1 and 2

15-i) Report demographics associated with digital divide issues

Table 1 and 2

16a) CONSORT: For each group, number of participants (denominator) included in each analysis and whether the analysis was by original assigned groups

16-i) Report multiple "denominators" and provide definitions

NA

16-ii) Primary analysis should be intent-to-treat

Like this: " All outcomes were compared between randomised groups under the intention -to -treat principle (that is, including all randomised individuals in their originally randomised groups). "

17a) CONSORT: For each primary and secondary outcome, results for each group, and the estimated effect size and its precision (such as 95% confidence interval)

Seen in table 3 and figure 2

17a-i) Presentation of process outcomes such as metrics of use and intensity of use

NA- singel session

17b) CONSORT: For binary outcomes, presentation of both absolute and relative effect sizes is recommended

NA

18) CONSORT: Results of any other analyses performed, including subgroup analyses and adjusted analyses, distinguishing pre-specified from exploratory

In the assessment for skewness of the continuous variables we found 1 outlier in the treatment group (with weekly alcohol consumption of 1044 g/week) and 2 outliers in the control group (with weekly alcohol consumption of 1128 and 1524 g/week). The maximum reported weekly alcohol consumption of those not excluded was 552 g/week in the treatment group, and 456 g/week in the control group. We therefore performed a sensitivity analysis without these outliers. In this analysis, which was not specified a priori, the between-group difference in the primary outcome, weekly alcohol consumption, in the primary adjusted analysis, crossed the conventional threshold for statistical significance
(8% reduction (95% CI 0% to 15% reduction, p=0.049 adjusted; 10% reduction (95% CI 1% to 17% reduction), p=.02 unadjusted)

18-i) Subgroup analysis of comparing only users

NA

19) CONSORT: All important harms or unintended effects in each group

NA

19-i) Include privacy breaches, technical problems

No technical problems in this study

19-ii) Include qualitative feedback from participants or observations from staff/researchers

NA

DISCUSSION

20) CONSORT: Trial limitations, addressing sources of potential bias, imprecision, multiplicity of analyses

20-i) Typical limitations in ehealth trials

Like this: We did not have statistical power to detect the effect size that we believed was worth obtaining in the planning of this study. We succeeded in recruiting only one quarter of our target sample size, and the best estimate of the effect obtained on the primary outcome (a 6% reduction in alcohol consumption) is of clear public health significance. For example, it is very close to the size of effects considered appropriate for the implementation of face-to-face brief
intervention programmes [25] and measures to increase the price of alcohol [26]. "

21) CONSORT: Generalisability (external validity, applicability) of the trial findings

21-i) Generalizability to other populations

Like this: The effects are also broadly similar, for example with THRIVE showing a 17% reduction in alcohol consumption in comparison with controls after one month, decreasing to 11% after 6 months [30]. Some effects among Maoris were somewhat larger, though effects among non-Maori in the parallel e-SBINZ trial were smaller [31-32]. AMADEUS-1 [16] found that feedback added little to the effects of assessment, and detailed investigations of intervention content are urgently needed, in order to ascertain whether it may be possible to develop novel interventions or intervention components capable of larger effects than have been identified to date. This under-development of online study is similar to that which pertains for face-to-face brief interventions [33].

21-ii) Discuss if there were elements in the RCT that would be different in a routine application setting

Our study used the same elements and procedure as routine provision of the interventions to students in Sweden

22) CONSORT: Interpretation consistent with results, balancing benefits and harms, and considering other relevant evidence

22-i) Restate study questions and summarize the answers suggested by the data, starting with primary outcomes and process outcomes (use)

Like this: Table 3 reveals, however, that this study does not rule out an intervention effect of up to 13% reduction in total weekly alcohol consumption. The sensitivity analysis excluding outliers suggests an intervention effect on reduced total weekly alcohol consumption, though the statistical significance attained in that analysis should be treated with caution, as the particular analysis was not pre-specified. We have no reason to anticipate later occurring effects, as brief intervention effects generally wane with time [5] and the short term nature of this evaluation study is important to note in interpreting study findings."

22-ii) Highlight unanswered new questions, suggest future research

Like this: "The existing evidence suggests that these types of alcohol policies are most likely to be effective and we do not know whether or how far individual- level interventions in whole populations such as that evaluated here may enhance the anticipated effects [36]. "

Other information

23) CONSORT: Registration number and name of trial registry

Trial Registration: ISRCTN02335307

24) CONSORT: Where the full trial protocol can be accessed, if available

Alcohol assessment & feedback by e-mail for university student hazardous and harmful drinkers: study protocol for the AMADEUS-2 randomised controlled trial. McCambridge J, Bendtsen M, Karlsson N, White IR, Bendtsen P. BMC Public Health. 2013 Oct 10;13:949. doi: 10.1186/1471-2458-13-949

25) CONSORT: Sources of funding and other support (such as supply of drugs), role of funders

The study was funded by the Swedish Council for Working Life and Social Research (FAS, in Swedish; Grant number 2010-0024) and by a Wellcome Trust Research Career Development fellowship in Basic Biomedical Science (WT086516MA) to JM. IW was supported by the Medical Research Council [Unit Programme number U105260558].

X26-i) Comment on ethics committee approval

Like this: " Ethical approval for the study was granted by the Regional Ethical Committee in Östergötland, Sweden, number: 2013/46-31. "

x26-ii) Outline informed consent procedures

Like this: " If eligible for trial participation, they were provided with information permitting informed consent, making the present study unlike AMADEUS--1. "

X26-iii) Safety and security procedures

NA

X27-i) State the relation of the study team towards the system being evaluated

Declaration of interest

PB and MB own a company that has developed the online intervention used in this study and that develops and distributes computerised lifestyle interventions.
